# Factors Influencing Parental and Individual COVID-19 Vaccine Decision Making in a Pediatric Network

**DOI:** 10.3390/vaccines10081277

**Published:** 2022-08-08

**Authors:** Angela K. Shen, Safa Browne, Tuhina Srivastava, Jeremy J. Michel, Andy S. L. Tan, Melanie L. Kornides

**Affiliations:** 1 Vaccine Education Center, Children’s Hospital of Philadelphia, Philadelphia, PA 19146, USA; 2 Leonard Davis Institute of Health Economics, University of Pennsylvania, Philadelphia, PA 19104, USA; 3 Department of Medical Bioethics and Health Policy, Perelman School of Medicine, University of Pennsylvania, Philadelphia, PA 19104, USA; 4 Department of Biostatistics, Epidemiology and Informatics, Perelman School of Medicine, University of Pennsylvania, Philadelphia, PA 19104, USA; 5 Department of Pediatrics, Perelman School of Medicine, University of Pennsylvania, Philadelphia, PA 19104, USA; 6 ECRI Guidelines Trust, ECRI, Plymouth Meeting, PA 19462, USA; 7 Annenberg School for Communication, University of Pennsylvania, Philadelphia, PA 19104, USA; 8 Department of Family and Community Health, School of Nursing, University of Pennsylvania, Philadelphia, PA 19104, USA; 9 Department of Pediatrics, Division of Adolescent Medicine, Perelman School of Medicine, University of Pennsylvania, Philadelphia, PA 19104, USA

**Keywords:** COVID-19, vaccine decision making, vaccine uptake

## Abstract

Aspects of the COVID-19 vaccination campaign differed from routine vaccines, including emergency use authorizations, the prioritization of access, and the politicization of messaging. Subsequently, many parents reported lower vaccine confidence relative to routine vaccines, and vaccination coverage stalled below targets. This study aimed to understand parental vaccine decision making and compare COVID-19 versus routine vaccine decision making. We conducted nine virtual focus groups between 25 February 2022–11 March 2022 with parents (*n* = 41) of the Children’s Hospital of Philadelphia’s patients, recruited via email and stratified by vaccine hesitancy status (non-hesitant vs. hesitant). Transcripts were analyzed using the vaccine hesitancy matrix domains. Of 41 total participants, 25 (61.0%) were non-hesitant, 16 (39.0%) were hesitant or their children were not up-to-date on adolescent vaccines, and most self-identified as female (95.1%) and White/Caucasian (61.0%). Most participants (87.5%) were fully vaccinated against COVID-19 and many of their first children (*n* = 26, 63.4%) were vaccinated against influenza. Several themes emerged regarding decision making: individual influences, group influences, vaccine and vaccine program influences, and contextual influences. While some influences were similar for routine and COVID-19 vaccine decision making (e.g., needing evidence-based information), other factors were vaccine- or situation-specific. Building trust requires a multi-faceted concerted effort that involves addressing the complex vaccine decision-making process.

## 1. Introduction

Vaccine decision making is complex, especially for parents deciding for their children rather than themselves [1,2,3,4]. Hesitancy to vaccinate is a major public health concern, and U.S. parents are hesitant about specific childhood vaccines [5]. For example, approximately 26% are hesitant about the influenza vaccine and 23% are hesitant about the human papillomavirus vaccine with concerns centered on vaccine side effects, the severity of the disease prevented by vaccines, and vaccine effectiveness [6,7]. Similarly, parents have the same concerns about COVID-19 vaccines [8,9]. Within the current COVID-19 pandemic, decision making has become even more complicated. Knowledge about the disease and the long-term impact on various populations groups including children is emerging in real time. Initial access to vaccines has been authorized under pandemic emergency provisions while pending licensure, and the pressing nature of the vaccination program to vaccinate as quickly and as equitably as possible has added pressure on individuals and parents to make decisions [10,11,12,13,14].

**Figure 1:**
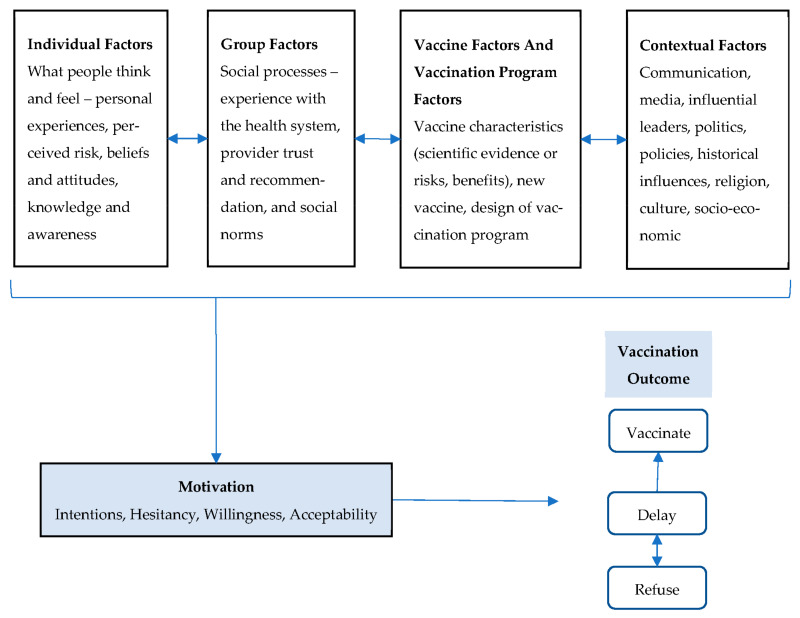
Factors influencing parental and individual vaccine decision making: COVID-19 and routine vaccines. Adapted from: WHO Vaccine Hesitancy Matrix developed by the Strategic Advisory Group of Experts on Immunization Vaccine Hesitancy Working Group (2015) [1], and Brewer, N.T.; Chapman, G.B.; Rothman, A.J.; Leask, J.; Kempe, A. Increasing vaccination: Putting psychological science into action. *Psychol. Sci. Public Interest* **2018**, *18* 149–207 [16].

These decisions are influenced by multiple factors including individual factors such as personal experiences, group level influences such as social norms, vaccine product specific characteristics such as the safety and efficacy profile of the vaccine, and attributes of the vaccination program design (i.e., mass vaccination) that are influenced by contextual factors including politics and policies [11,12,15,16]. These factors influence parent’s motivation and intentions to vaccinate (e.g., making an appointment) and ultimately the decision to vaccinate or delay vaccination (Figure 1) [14,15,16].

In this study, we explored parental values, beliefs, and attitudes relative to vaccine decision making for routine and COVID-19 vaccines to better understand the important factors of each at a time when children younger than five years of age could not be vaccinated against COVID-19 and vaccination rates of those older than five years fell below intended pandemic targets. Continuing to improve our understanding of this decision-making process is critical for making parents feel supported in their decision-making process and ultimately achieving broader vaccination coverage for all vaccines.

## 2. Materials and Methods

### 2.1. Study Design, Participants, and Setting

We conducted a qualitative study among families using the Children’s Hospital of Philadelphia (CHOP) care outpatient primary care system, which consists of 31 primary care sites located within the greater Philadelphia region. Sites are designated as urban academic (*n* = 3, located in Philadelphia, PA, USA), urban non-academic (*n* = 3), or suburban (*n* = 25, 22 in PA and 3 in NJ) (Supplemental Table S1).

Using the CHOP electronic health record (Epic Systems, Inc., Verona, WI, USA), we identified 40,583 eligible families using the following criteria: (1) if they had at least one living child between birth and 19 years of age, (2) if the child had received care between 1 January 2019 and 30 September 2021, and (3) if they had not opted out of all research. These families were invited to complete two sequential online surveys (October 2021–February 2022) about COVID-19 vaccination among children. Families who completed both surveys were subsequently invited to participate in the study described herein and stratified by hesitancy status using the Helmkamp scale (Supplemental Figure S1) [17].

To make our hesitant cohort more robust, we also identified 5230 parents of adolescents from the original 40,583 cohort whose children were not up-to-date (UTD) with vaccines routinely recommended for adolescents as of 24 February 2022, as specified by the Immunization for Adolescents Healthcare Effectiveness Data and Information Set (HEDIS^®^) measure (Supplemental Figure S1) [18]. A randomized subset of families from this group was also invited to participate in this study and were considered hesitant for the purposes of this study.

We conducted nine one-hour virtual video focus groups (FGs) of 3–6 participants each from 25 February 2022 to 11 March 2022. Five FGs were conducted with non-hesitant parents, and four were conducted with parents of adolescent non-UTD or hesitant parents, as determined by the Helmkamp scale to stratify parents by hesitancy status [17]. Of the 80 invited to participate, 46 participated (response rate = 57.5%). Information on demographics, acceptance of routine vaccines, COVID-19 vaccination status and intentions, perceptions about COVID-19, access to vaccination services, COVID-19 anxiety, and the use of social media was collected from all participants in advance of their FG. This study was approved by the CHOP Institutional Review Board. All participants completed informed consent documentation. 

### 2.2. Data Collection

A semi-structured interview guide was developed from previous COVID-19 vaccine hesitancy research [19,20,21]. Question domains included reasons for or against vaccination, social norms, information sources, access to services, and recommendations for improving access (Table 1). During the discussions, participants were instructed to consider their decision-making process for vaccination of themselves (individual) as well as of their children (parental).

### 2.3. Data Analysis

FG interviews were recorded and transcribed using a transcription service. We analyzed transcripts and interview notes using a thematic approach, and findings were reported using the Standards for Reporting Qualitative Research (SRQR) reporting guidelines [22,23,24]. Two experienced coders (AKS and SB) reviewed the transcripts and field notes to develop a preliminary codebook and then tested and amended this codebook after coding two transcripts. Coders reached iterative consensus on the codebook, code definitions, and coding approach, and used memos to document the thematic evolution in the analysis. Triangulation was achieved with iterative discussions that included all moderators and facilitators.

Frameworks developed by the World Health Organization’s vaccine hesitancy matrix (VHM) were modified based on current psychological science [1,3,15,16,25]. Themes were categorized into four determinant domains, as illustrated in Figure 1: individual factors, group factors, vaccine-specific or vaccination-program-related factors, and contextual factors. This approach allowed for a multi-faceted consideration of vaccine intent, as well as motivation and intended behaviors for both routine- and situation-specific vaccines in the context of a dynamic environment (e.g., evolving pandemic and response).

## 3. Results

Nine focus groups (FGs) with 41 total participants (95% female) were conducted with 25 non-hesitant participants and 16 hesitant non-UTD participants. Non-hesitant participants were largely White or Caucasian (80%, *p* = 0.006) and mostly had household incomes over USD 75,000/year (84%). Regardless of hesitancy status, most participants (68.3%) were fully vaccinated against COVID-19 (Table 2). Participants described factors that influenced their decision making for routine and COVID-19 vaccinations. Figure 1 shows themes organized by domain, and Table 3 shows the relevant quotes. Notably, the results reflect an inter-relatedness of factors that influence decision making. 

Participants stated a clear differentiation between decisions to act for their themselves as adults and decisions for their children. Participants in two hesitant non-UTD groups echoed this sentiment on delay and hesitation: “I was scared to get it because of the unknown, because the vaccine hadn’t been out for much, but I trust the science and I did that for my kids as well, but I did delay their COVID vaccines for a bit. I didn’t get it as soon as it was available for them” (FG 6) and “I mean because making a decision for myself, I can live with the consequences I chose to whatever it is, but when it comes to my children, if I make the wrong decision, that will weight a lot more heavily on me” (FG 8).

When asked how participants currently (February/March 2022) feel about the pandemic, they reported two polar sentiments: either being “tired, exhausted, over it, frustrated, sad, challenged” or being “accepting, optimistic, and hopeful about the pandemic”. During the interviews, participants reported divisive experiences in their lives around vaccination with sentiments falling into three general categories. FG 1 primarily focused on practical issues of getting vaccinated while other groups (FGs 2–4) focused on the altruistic motive for vaccinating to help others for the greater good. Conversely, the hesitant non-UTD cohorts (FGs 5–9) were torn between tacitly encouraging vaccination and expressing the sentiment that individuals should not push their opinions on others.

### 3.1. Individual Factors

#### 3.1.1. Personal Medical History and Experience with Previous Disease

Many participants described their concern with pre-existing and co-morbid conditions (e.g., asthma, autism), as well as previous experience with vaccine-preventable diseases (VPD), specifically COVID-19 disease and cervical cancer, as compelling reasons for vaccination. Participants described how personal experiences and seeing cases of VPDs, such as measles, and babies suffering are “horrible” and that they “didn’t want to go through that [COVID] and I don’t want my kids to go through that” (FG 2). Some participants also cited not vaccinating for a specific VPD because they were already immune from having had the disease (e.g., chickenpox) or because they survived the disease (i.e., influenza).

#### 3.1.2. Beliefs (Autism, Altruism, and Conspiracy)

Participant beliefs, such as the belief that vaccines caused their child’s autism, the obligation to protect others for the greater good, and government conspiracies, influence their decisions (Table 3). One participant stated “play your part by getting them [children] vaccinated too. And that’s how you keep mumps away from everybody… and I did not have any concerns for us for COVID by myself… and I did not have any concerns [for my children]” (FG2). Participants perceived that their children were more vulnerable than themselves, with younger children being most vulnerable. A few participants felt that COVID “is a manmade disease… and I just don’t want to play Russian roulette with my kids’ life [and vaccinate]” (FG 6).

#### 3.1.3. Knowledge to Make Decisions Is Informed by Trusted Sources

Participants felt that having trusted information sources, specifically trained experts, was central to their decision making and empowered them to be able to make informed decisions by having the facts that they needed (e.g., technology and science behind the vaccine). Participants were aware of the disease and the vaccine, but they stated that they were not experts and needed to hear from experts. Participants emphasized the autonomy of their decision making. In addition, many lost their trust in government sources, specifically the Centers for Disease Control and Prevention (CDC). They indicated that while the CDC was previously a trusted source, they were now perceived as bowing to political pressure during the pandemic.

#### 3.1.4. Risk Perception of Disease as Burden Shifts

As COVID-19 case counts and hospitalizations shifted, participants described how their risk perceptions changed. When community transmission and case counts were high, participants felt that there was a compelling reason to vaccinate because “the side effects and the potential long-term side effects of actually having COVID can or are known and perceived to be worse” (FG 9). While participants were aware of the disease and the availability of the vaccine, they described that the most difficult part of their decision making was the time pressure they felt to decide about vaccination given their community’s disease burden. This time pressure was contrasted to decisions about routine vaccinations, stating that those vaccines have “been around for a long time” and the burden of disease for other VPDs is low.

### 3.2. Group Factors

#### 3.2.1. Provider Trust and Experience with Health System

Many participants emphasized trust in their health system (e.g., CHOP for their children, Veterans Affairs for themselves) and in their child’s pediatrician and their own doctor, particularly participants with pre-existing or co-morbid conditions. Provider recommendations were often cited as the most compelling source for deciding to vaccinate: “I trust what my doctors are telling me that my kids need” (FG 5). Participants also cited their trust in and respect for family, friends, and colleagues with expertise in the health field, including those who work at pharmaceutical companies.

#### 3.2.2. Vaccination as a Norm and Social Norm

Many participants described the routine vaccination schedule as a norm strengthened by the fact that vaccines are required for school entry. Participants often described how their parents or they were vaccinated, and that it was “just done”. Participants cited exceptions to the norm, particularly influenza and newer vaccines such as HPV, as the “only one” which parents out opt of giving to their children.

### 3.3. Vaccine and Vaccination Program Factors

#### 3.3.1. Scientific Evidence of Risk and Benefit: Long-Term Safety and Efficacy

Many participants pointed out that while short-term safety can be detected by waiting fifteen minutes at the vaccination clinic, the unknowns about long-term safety were worrisome. Participants in FG 8 stated “I’ll be the test dummy. I’ll be the first one to get it before I consider getting my children vaccinated” and “the difficulty in making a decision about vaccines is obviously whether or not your child could be the one that could potentially suffer an adverse event”.

#### 3.3.2. “Newness” of the Vaccine

Participants noted how new the vaccine was and contrasted the rapid introduction of this vaccine to routine vaccines. They described their anxiety around the “newness” of the vaccine and of the disease, though some participants felt COVID-19 disease has been around for a long time.

#### 3.3.3. Vaccination Program Design and Supply

Most participants indicated a preference to receive vaccines in a medical setting to ensure that adverse events could be appropriately managed. Many parents stated preferences for their children to be vaccinated in their pediatricians’ offices, but they were fine with receiving vaccinations themselves in pharmacies and other settings (e.g., occupational health). “I don’t believe in shots from CVS… we don’t do anything in pharmacies in Poland, other than taking your prescription drugs from a pharmacy. I don’t really get flu shots at CVS. I can buy a greeting card at CVS and refill my prescription, but not necessarily having someone put a needle in my body… it’s just like a cultural thing” (FG 1).

Some participants found COVID-19 vaccine appointments with ease while others had frustrating experiences. While participants who wanted to vaccinate right away actively sought earlier appointments over their location of preference, most cited a desire to receive vaccination services from providers they knew and trusted. Having a choice of vaccine brand was also important to some participants, particularly given reports of some adverse vaccine candidates.

### 3.4. Contextual Factors

#### 3.4.1. Communication and Media

Communication about the near-real-time events unfolding during the pandemic made communicating about the disease and the importance of vaccination more complicated. While participants cited media as a source of information, they also described how hearing about stories from others (about routine or COVID-19 vaccines) signaled to them that there must be something to be cautious about because people are talking about it. Specifically, with COVID vaccines, participants cited confusion around the Food and Drug Administration (FDA) authorization for children under age five years and wished for clarity and transparency from the credible sources they use. Some participants, but not all, stated that they sought information about COVID-19 vaccines from different sources than for routine vaccines; their primary care provider for routine vaccines was their first line source for information whereas they used additional or other sources for COVID including the health department or news outlets.

#### 3.4.2. Politics and Policies

Many participants described their frustration and fatigue related to politics and partisan divide: “I mean, I’m sure there are people who are Republican, who do get vaccinated and Democrats who don’t get vaccinated. But, it’s just like, everything is like black or white. It’s like this or that. And there’s no, there’s nothing in between… there’s no room for the gray... And if you try to get somewhere in between, like no one wants to hear from you ever again ….” (FG 5).

During the interviews, many participants indicated that they work in healthcare. When mandates were discussed, participants voiced mixed sentiments. Some people did not like being told what to do, yet others felt mandates were necessary to protect their children, themselves, and their families and communities. Likewise, some participants explained that they were vaccinated for routine vaccines because it is a school entry requirement. However, others indicated that COVID-19 mandates pushed them away from accepting vaccination, even if they intended to do so previously.

#### 3.4.3. Historic Influences, Religion, Color, Gender, and Socio-Economic Status

Some participants cited historic distrust of the government. For example, one participant stated “you can’t really go back in time and undo Tuskegee, so I think there’s always going to be some kind of uphill battle [to vaccinate]… due to the history of the United States, there’s always going to be people who are just distrustful of the government and in some cases rightfully so. I don’t know how to convince those people [to vaccinate].” Others indicated that their religion helped drive their choices. As explained by one participant, “…we just pray for peace and protection, each day, move forward in that way” (FG 9).

### 3.5. Guidance for Other Parents and Recommendations to Policymakers

Participants offered guidance for other parents about COVID-19 vaccination and for policy-makers regarding ways to improve trust, confidence, accessibility, and communication with parents (Table 4). Guidance for other parents centered around taking ownership and carrying out evidenced-based research. Participants emphasized the importance of sharing their own personal experiences, communicating with empathy, acknowledging and validating others’ feelings, and allowing people the opportunity to ask questions while conveying that vaccination prevents serious harm and that their actions affect others. Guidance to policy-makers was centered around apolitical, transparent, and honest communication on policies and practices with deep consideration policies such as removing mandates.

Participants felt that having respect for other people’s beliefs and opinions is fundamental to talking to other parents, as illustrated in two participants views here from the hesitant and non-UTD cohort: “I would prefer people to be vaccinated by I’m accepting of their choice to not be in an uncomfortable with that.” (FG9) and “Ultimately, I think we all need to feel that we made the right decisions for our kids, whatever that is. Not all kids can get the vaccine safely. So I just think, you know, do your homework and do what you’re comfortable with” (FG9).

Participants also noted the freedom and empowerment associated with having some control during the pandemic. As one participant stated, “So again, it’s not a hundred percent blanket of, you know, not getting it, but just for protecting myself and other people, It’s just a freedom, you know, that choice. …you have the mask, you can wear masks around people who are not vaccinated, whether they’re vaccinated or not vaccinated yet” (FG 9).

## 4. Discussion

Above all, participants clearly wanted their children to be safe amidst the pandemic and struggled to make the vaccine decision for their children. The weight of these decisions involved multiple inputs with different risk tolerance for themselves as compared to their children. Concerns and trust in their primary care provider (typically their child’s pediatrician) were common to hesitant and non-hesitant parents. In weighing risks of the disease versus the vaccine, parents described struggling because of vaccine safety concerns, newness of both the disease and the vaccine, and a sense of time pressure to make their decision given the risk of getting sick. Personal experience with VPDs, particularly COVID-19, affected the amount of risk individuals who were willing to vaccinate their children. All together, these inputs were bounded by the decision to vaccinate quickly, a concern often mitigated by a licensed vaccine (versus emergency authorized) and time on the commercial market (to provide an increased confidence in safety and effectiveness).

Changing community transmission led many to accept vaccination, particularly those at increased risk, and given the decline in routine VPDs, an individual’s perceived risk matters. The changing rates of community transmission, coupled with increased access to and expanded age recommendations for COVID-19 vaccines, were discussed with increased sensitivity by respondents. Although routine vaccine coverage has declined because of low perceived risk, participants perceived their risk of COVID-19 as high; therefore, they tended to accept vaccination even if they were nervous about doing so. Recognizing levels of risk tolerance can inform how messages are shaped to parents. It is clear the higher the case counts, the more inclined individuals who may have been “on the fence” may have decided to vaccinate. Moreover, the autonomy to make this decision to protect oneself and one’s family—through masking, social distancing, or vaccination—provides some sense of control at a time where many feel helpless in the throes of a pandemic.

Messaging about vaccination is complex. There are situational reasons (e.g., a developing science that the public does not understand), the role of the media (which can be different than the goals of scientists), and the political and social ramifications of the pandemic (e.g., mandates, inequities in access) which should be considered. Politicization of the response to the pandemic was clearly evident and emphasized by respondents. Views on vaccines, vaccinations, and other interventions (such as masking) were documented early on in the response along party lines [26]. Vaccination has been controversial for centuries, but in the U.S., only in this pandemic has political affiliation so greatly impacted disease and vaccine attitudes and behaviors. COVID-19’s transmissibility and unknown manifestations led to nationwide quarantine and shut-down policies, a high number of deaths and hospitalizations, and a spread of fear (including the implications of long COVID) [27]. A combination of these reasons contributed to the systematic failure of public health messaging and has led to a confused and frustrated public. This has made it even more difficult for some to trust the public health system. Primary care providers such as trusted advisors have helped decision-makers to navigate the confusion, alongside the emerging concern about the long-term impact on the mental health of our communities, particularly in children [28]. Investing in training these providers on how to communicate with parents may be critical to raising coverage.

Focus group discussions illustrated that a one-size-fits-all view about population behavior can lead to false assumptions (e.g., all health care workers are pro-COVID vaccine) [29]. Beliefs, personal experiences, and situational context affect vaccine decision making. As such, those who receive the same information will process it differently. Recognizing this heterogeneity should be considered in tailoring public health strategies and communication. Most importantly, vaccine decision making is complex; therefore, approaches to those who have questions must be empathetic and must seek to understand their concerns on an individual basis—even at an individual point in time or for an individual vaccine—rather than being tempted to apply a one-size-fits-all response that applies a knowledge deficit model of engagement that simply communicates information about vaccine safety and efficacy [30,31,32].

### Limitations

Our study had the following limitations: (1) recruitment in the hesitant cohort was low; thus, we expanded inclusion criteria to include parents of children who were not UTD with their adolescent vaccines (with HPV as a common driver of hesitancy); (2) we did not exclude parents who were employees of CHOP or worked in the medical or public health field, which may have biased our sample to more informed participants; (3) our findings may not be generalizable to other patient populations or geographic areas; and (4) virtual participation may have led to a selection bias excluding individuals with limited telephone or internet access.

Notably, the response rate to the focus group invitations was low, possibly reflective of a COVID environment where there is a saturation of information requests coupled with the challenges of managing life during a pandemic [33]. Despite this, the thematic saturation of the interviews provided insights across the study period hinged on the dynamic nature of the pandemic and the government’s response. The low participation of males is not uncommon as matriarchs of the family are generally more engaged in health promotion behavior and make many health care decisions, limiting how much gender differences contribute to the overall bias in this study [34,35].

## 5. Conclusions

By necessity, the scientific and public health response to COVID-19 unfolded quickly, leaving individuals and families with little time to process the changing situation and gain understanding of new information amidst a global public health emergency. Even for routine immunizations, vaccine decision making is complex, but during the COVID-19 pandemic, this decision making was further complicated. The newness of the disease and the vaccine, political effects on public health decision making, time pressure, and a sense of loss related to individual autonomy explain why individuals were more hesitant to comply, even when confronted with messaging related to the broader need to reach community immunity. As demonstrated by these focus groups, the need to protect the entire community, even during a public health emergency, involves the necessity of approaching the individual and taking the time to understand what considerations are going into their personal vaccine decision making through transparent apolitical engagement with respect for the concerns of communities and autonomy in decision making.

## Figures and Tables

**Table 1:** Focus group question guide.

Ice breaker. Can you tell us your first name, how many kids do you have, and how old they are? In a word or two, how do you feel about the pandemic right now? Reasons for Vaccination Why do you choose to vaccinate (or not vaccinate) your kids for routinely recommended vaccines like tetanus, measles, and pneumonia? What concerns do you have about vaccinating your kids with routinely recommended vaccines? What additional information do (did) you need to feel comfortable with vaccinating your kids with routine vaccines? Why did you choose (or not choose) to get the COVID-19 vaccine for yourself? For your kids? What concerns do you have about COVID-19 vaccination for yourself? For your kids? What additional information do (did) you need to feel comfortable to vaccinate and is there a difference in your information needs for the decisions you make for yourself versus your kids? Social Norms (COVID-19) How comfortable are you around people who are not vaccinated? What if they are family and friends? What would you do around other people who you know are not vaccinated? How comfortable are you with your child being in school with others who are unvaccinated and with the steps your child’s school is taking to protect your child? Information Sources Specifically, thinking about how you make decisions about vaccinating your kids, from where or whom do you receive information about vaccines? Could you identify one source you trust the most in shaping your decisions? Why? Does this information source differ between routine and COVID-19? What makes it easy (or difficult) to make decisions about vaccination? Access From whom and where would you feel most comfortable getting vaccination services? How easy was it to get a COVID-19 vaccination for yourself and for your kids? Closing What would you say to other parents in Philadelphia and your community about getting COVID-19 vaccines for themselves and for their children? What would you say to policy makers about how to improve trust, confidence, accessibility, and communication to other parents?

**Table 2:** Baseline sociodemographic and vaccination characteristics of focus group participants (N = 41) by vaccine hesitancy status.

Characteristic	Non-Hesitant (N = 25)		Hesitant (N = 16)		*p*-Value
**Age**, median years [IQR]	40 [35–45]		46.5 [42–49]		0.004 *
	**N**	**%**	**N**	**%**	
**Sex, assigned at birth**					0.512
Female	23	92%	16	100%	
Male	2	8%	-	-	
**Gender**					0.700
Woman	22	88%	16	100%	
Man	2	8%	-	-	
Non-binary	1	4%	-	-	
**Sexual orientation**					0.266
Heterosexual or straight	19	76%	15	94%	
Gay or lesbian	-	-	1	6%	
Bisexual	3	12%	-	-	
Queer	1	4%	-	-	
Prefer not to answer	2	8%	-	-	
**Hispanic, Latino/a/x, or Spanish origin**					
Yes	1	4%	-	-	-
No	24	96%	16	100%	
**Race**					
White or Caucasian	20	80%	5	31.3%	0.006 *
Black or African-American	3	12%	9	56.3%	0.014 *
Asian	1	4%	1	6.3%	
Multiple	1	4%	1	6.3%	
**Household income**					0.085
Less than USD 25,000/year	1	4%	2	12.5%	
USD 25,000–49,999/year	2	8%	3	18.8%	
USD 50,000–74,999/year	1	4%	3	18.8%	
USD 75,000–99,999/year	4	16%	2	12.5%	
USD 100,000–149,999/year	4	16%	4	25.0%	
Over USD 150,000/year	13	52%	2	12.5%	
**Highest degree**					0.048 *
Regular high school diploma	-	-	2	12.5%	
GED or alternative credential	-	-	1	6.3%	
Some college credit	2	8%	6	37.5%	
Associate’s degree (i.e., AA, AS)	2	8%	-	-	
Bachelor’s degree (i.e., BA, BS)	5	20%	3	18.8%	
Master’s degree (i.e., MA, MS, MSW, MBA)	10	40%	2	12.5%	
Professional degree (i.e., MD, DOS, JD)	3	12%	1	6.3%	
Doctorate degree (i.e., PhD, EdD)	3	12%	1	6.3%	
**Number of children**					0.187
1	12	48%	4	25%	
2	9	36%	4	25%	
3	3	12%	5	31.3%	
4	1	4%	2	12.5%	
6	-	-	1	6.3%	
**COVID-19 vaccination status, parent**					
Yes, fully vaccinated	24	96%	14	87.5%	0.550
No	1	4%	2	12.5%	
**Influenza vaccination status, parent**					0.554
Yes	20	80%	11	68.8%	
No, but I intend to	-	-	1	6.3%	
No	5	20%	4	25%	
**Influenza vaccination status, first child**					0.043 *
Yes	19	76%	7	43.8%	
No, but I intend to	-	-	2	4.9%	
No	6	24%	7	43.8%	

IQR = inter-quartile range. * indicates significant *p*-value (α = 0.05). Note: For all categories except age, for which a *t*-test was used, Fisher’s exact tests were used to determine *p*-values.

**Table 3:** Themes and illustrative quotes on factors influencing parental vaccine decision making.

Theme	Quotes
**Individual Factors**	
Personal medical history and experience with previous disease	“So I want her [daughter] vaccinated ASAP. She has asthma, She had RSV over the summer. I’ve already seen her on oxygen before.” (FG 1: non-hesitant)
	“My grandmother had polio in 1952 she got it and she was in an iron lung for about nine months…. My parents noted like this is why we get the vaccinations because look what happened to grandma.” (FG 2: non-hesitant)
	“I also lost friends to COVID early on and colleagues and it was honestly home-grown videos that people were post-ing that really got me there and recognize that if I were to get it and I passed away, what that would do and look like for my family and for my kids.” (FG 4: non-hesitant)
	“When the kids were younger, we were really good about getting the flu shot and then we knew the strains could be different. And my daughter got the flu anyway, and I think we got lax after that.” (FG 7: hesitant non-UTD)
Beliefs (altruism, conspiracy, autism)	“My son has autism and intellectual disability. I have questioned whether or not I should have got him vaccinated, when he got vaccinated. I had some concern after he developed his disabilities about the amount of vaccinations that he was receiving at one time. However, despite those beliefs that I had, I still chose to get him vaccinated against COVID.” (FG5: non-hesitant)
	“I just felt like the whole COVID situation itself, like I said, was just like a conspiracy to de-population. So I don’t re-ally trust anything that has to like deal with it for real for real.” (FG 6: hesitant non-UTD)
Knowledge (facts) to make decisions is informed by trusted sources	“When a Lyme disease vaccine became available for people, I was one of the first, I was so excited. I went out, I did it. And then as time happened, it stopped being a recommended thing. So that’s why I don’t do a blanket. I will do it. Sometimes the science catches up. But to the best of my ability and with the information that’s available at the time I have to make a decision, I will make that decision. I’m just not going to blanket say whatever it is that’s recommended I will do.” (FG 1: non-hesitant)
	“…the part that is difficult to me as a parent, to be honest is even though I was feeling like 99% good about it, I always feel like it’s hard to choose something for your child’s body. Like it’s easy to choose for yourself.” (FG 2: non-hesitant)
	“As far as in a COVID shot or whatever, I just feel like, give me a couple of more years and see what had happened. Like, I felt like it was something that was made too fast. It’s not too many studies, you know, it’s only been a couple of years into it. Somewhere down the line it might be an underlining situation. Underlying situation, meaning something will come up that we didn’t know before.” (FG 6: hesitant non-UTD)
	“I think my doctor’s office told me they had never seen any kind of adverse effects in their practice. It’s a lot. You know, as a large [medical] group it just kinda helped. They gave me a little bit of a history of the vaccine [and] talking to my kids the doctors did with me present about what are they risking by not getting the vaccine at that point [helped]” (FG 9: hesitant non-UTD)
Risk perception of disease as burden shifts	“I’m super cautious because I’ve seen what it could do the extreme of deaths or whatever. I’ve seen personally, what it can do and it’s not something that somebody I feel should be laxed about.” (FG 8: hesitant non-UTD)
	“I thought that given the rates of serious illness and all of the deaths that were happening in a very short period of time, I thought it made sense, in my opinion, or on the side of caution and go with [vaccination.]” (FG 9: hesitant non-UTD)
**Group Factors**	
Experience with health system and provider trust	“I don’t have much of a medical background…I don’t really understand how my lights work in my house, but I still use them” (FG 3: non-hesitant)
	“I’m a firm believer in partnering with my pediatrician. I place a lot of trust in them and believe that they’re going to make the right decisions. They’re the experts here.” (FG 8: hesitant non-UTD)
	“Oh, the vaccine itself and the way, how the government just put everything out…you know, that was the only concern that I really have. Like if someone’s vaccinated, I’m not like scared to be around them or anything like that. You know, I know nothing could come from it. It’s just there. I don’t know what they’re offering to put into our bodies” (FG 6: hesitant non-UTD)
	“I definitely don’t trust social media with anything. And, with news not being so on the up and up lately, I just don’t trust it anymore either. So I’m only going to trust the doctors. You pretty much get the same information from them, whether it’s routine vaccinations or with the COVID.” (FG 8: hesitant non-UTD)
Vaccination as a norm and social norms	“They’re [children] doing the best that they can, but their peer group with a lot of unvaccinated people, there’s a social component that I have to be comfortable with in order for their emotional wellbeing and a lot of their, those unvaccinated kids have had COVID. So at this point, they’re as immune as my kids…. We just went to an indoor playground…and I was crawling out of my skin with the people around us, but for my kids’ emotional wellbeing, I’m ready to make that phase back into society.” (FG 1: non-hesitant)
	“One thing I have used to convince some friends who are vaccine hesitant is I found something on Reddit, the Herman Cane award where people take photos of people on Facebook who were anti vax and got sick with COVID and died and on their death bed were like I wish I got the vaccine…. Because I think if you don’t know anyone who has died with COVID, you are probably less likely to take it seriously. Empathy is probably key to get someone to get vaccinated, so conveying it’s really serious and people are really dying.” (FG 3: non-hesitant)
	“My kids definitely get them [routine vaccines]. I do feel as though they [are] very important because I had them and it did no harm to me. You know what I’m saying? It was something that I experienced first in my lifetime, and know that it’s safe for my children. I have no concerns about vaccinating [for the COVID shot]..” (FG 6: hesitant non-UTD)
	“So high school level, a lot of people interpret that as I don’t have to wear a mask, even if my mom wants me to kind of thing. The policy that was in place was not at all protective and it was a very bullying environment. As, as one of the other moms said, it’s [school policies have] been a bullying environment this whole time making people feel bad for wanting to protect themselves or their families” (FG 9: hesitant non-UTD)
	“Yeah, well, the problem was it wasn’t like, I knew that a portion of our school population was not vaccinated, but I think for me, the problem was that people were knowingly sending their kids to school with COVID. They were knowingly sending their kids to school symptomatic and they were not masking. So it was like, they were almost guaranteeing the spread of COVID, to a point where it was ridiculous. It wasn’t like a normal. Like it was just, wasn’t normal. It wasn’t like going to the grocery store where everyone’s just masked and like, careful” (FG 9: hesitant non-UTD)
	“Itt just felt like the right thing to do, honestly. I didn’t really question any of it. You know, you read stories of history of what these horrible diseases have done. And if we have a way to prevent them, it just wasn’t something that, other than Gardasil, that was one that I did kind of put a little extra thought into and give it a little extra time, just because it was fairly new. But for the other stuff [routine vaccination], it wasn’t even a question for me.” (FG 9: hesitant non-UTD)
**Vaccine And Vaccination Program Factors**	
Scientific evidence of risk and benefit: long-term safety and efficacy	“…if you decide not to get the mumps vaccine, your kids still probably not going to get mumps because everybody else has already had the mumps vaccine and mumps isn’t going around right now. But COVID-19 is so even if you’re feeling like, oh, I wish I had more time, you know, you don’t have more time.” (FG 2: non-hesitant)
	“I think for us, it’s a lot of, you know, a lot of concern around what the long-term picture is…”. “I mean, we know a little bit about, like multi-system inflammatory syndrome in children…obviously we only have like a couple of years of data on this, so far, but for us really, it’s just the concern on the long-term effects.” (FG 4: non-hesitant)
	“I did get their vaccines, their COVID vaccines, but I did delay them for a few months just to see, you know, what adverse reactions were there. And I feel, you know, the same, I trust science. You know, even though I am still weary, I’m just going to have faith that, you know, it’s going to be a positive thing and we’re going to eliminate this virus and move forward.” (FG 6: hesitant non-UTD)
“Newness” of the vaccine	“I think COVID because there’s just so much different information, healthcare routines. I was like, we have kids, this is the schedule, we’re doing the schedule [for routine]. I never really thought twice about it. In fact, I think COVID because it’s quote unquote new, but I know it’s not entirely new” (FG 2: non-hesitant)
	“I felt like it was important to do it..., I don’t know if I want to be the first in line” (FG 3: non-hesitant)
	“I’m pretty comfortable with, I mean like chicken pox and all that have been out, you know, that vaccine has been out forever. I don’t hear anything, you know, bad about it. And as far as reactions and things like that with COVID, I’ve heard many different stories, whether it’s from patients, doctors, personal experiences. So it makes it a little iffy. Is it because they’re different, they’re conflicting information or just hearing about it, like hearing that there are reactions and things” (FG 7: hesitant non-UTD)
	“I just feel as though it hasn’t been out long enough, I would like more research to be done on it. Especially with my oldest having a heart murmur, they were saying something about it does it can affect heart in some adolescent children. So that is one concern. Other than it not being out long enough, I believe as time goes on, you know, they have to tweak medications and things like that” (FG 7: hesitant non-UTD)
	“I have two boys and a girl and I was honestly a little more hesitant with my daughter. She was the last one I vaccinated just because I was concerned about reproductive issues and the unknowns” (FG 9: hesitant non-UTD)
Vaccination program design and supply	“One thing that I will say is I was actually really disappointed that CHOP didn’t do a better job of reaching out to families to get them appointments… I had friends who had their…pediatricians who gave them appointments to be ready on that day. And CHOP didn’t have any for like two or three weeks. And they also didn’t offer a flu shot together with the COVID shot, which I also thought was a huge missed opportunity” (FG 4: non-hesitant)
	“So, I mean, if I could get it at the doctor, I would, but I felt so passionate about getting it quickly that I did it at a drive-up site that looked like a military operation in northern New Jersey” (FG 4: non-hesitant)
	“The access, maybe they should have more things out there because not everybody is available during normal business hours. So I was thinking maybe something where it could be more of a 24 h thing. You’ve got parents that might work overnight and you can’t get there in the timeframe to that opening” (FG 7: hesitant non-UTD)
	“In the beginning it was hard to get an appointment. But then, once you got your first appointment, they scheduled you for your second, and then the booster was fine” (FG 7: hesitant non-UTD)
	“The county made that easy. Actually you could just go, there were no appointments” (FG 7: hesitant non-UTD)
**Contextual Factors**	
Communication and media	“I think it’s a very sensitive topic, but when it comes to like, just protecting my friends, you know, adults, I do try to emphasize that there is a tremendous amount of data available around the safety and effectiveness of the vaccines. And that many times what they’re reading on, for example, social media is not, I hate to say this, but it’s, if they’re reading fake news, consider your sources and be sure that you’re not blindly clicking on some headline that when you dig deeper, this actually happened” (FG 8: hesitant non-UTD)
Politics and policies	“Originally for us it [the trusted source] was the CDC, but lately they’ve chosen not to follow the science and bow to political pressure. And so I am less inclined now to look at the CDC… because the politics, the pressure that’s coming from the public is, look, we’re getting ready to go into midterm elections and people are fed up with COVID and we just want to get back to normal and they’re sick of masks… And there really isn’t good reason to explain why they’re changing their position at this point, except for the pushback they are getting” (FG 1: non-hesitant)
	“I’ve lost a lot of respect for them [CDC], you know, at some point I did work for them. And so, I just feel like it was just a really botched rollout for various reasons. And a lot of the messaging was, even just about, even when you lost the messaging for the disease, it’s hard to really push for the vaccine.” (FG 5: non-hesitant)
	“And she said, oh, well, I was going to get it anyway. But I just don’t like being told what to do…. So I feel like when we start telling people that they have to, it just makes them push back even more against it”. (FG 3: non-hesitant)
	“I thought they should still be masked because kids started catching it a lot more and spreading it more freely than the adults were by the time they was opening up the school. So I didn’t like how parents wanted their kids unmasked because a lot of students, if you talk to them, there’s, they’re more afraid than the adults are”. (FG 8: hesitant non-UTD)
	“So I am, I think they’ve done a good job with it [school policies]. They’ve kind of taken a middle road. I think it’s being respectful of what the CDC is [recommending]. I think it’s following public health in a framework that, is respectful of personal choice within the CDC framework”. (FG 9: hesitant non-UTD)
	“Our school district, I would say I’m highly disappointed in our school district because it [masking policies] became political very quickly and they departed from CDC recommendations immediately. And it stayed on a course of, trying to get the masks out of the schools, trying to allow people to not wear masks, not promote promoting vaccinations. It became very, very ugly and even violent at times in our school district. So it just was kind of a disaster, I would say the whole thing. I’m not in agreement at all of how everything played out in my [school] district”. (FG 9: hesitant non-UTD)
Historic influences, religion, color, and gender, SES	“…but there’s still, you know, some trust issues there that don’t seem to be going anywhere anytime soon issues with the system or the providers or the recommendations. I think it’s a mix of the system and the providers and just being in tune to historical traumas that have happened and that are real and thinking about, okay, was this vaccine tested on black women…? And then we had the information about J and J and the blood clots and that was like a real thing… And so that’s been just a real conversation and hasn’t necessarily helped make these difficult decisions”. (FG 4: non-hesitant)
	“I had faith in the clinical trial results and I had faith in the process. I mean I probably could have had more faith in the process because I think the government there’s always issues with the government, in ineptitude”. (FG 9: non-UTD)
	“You know, I, there’s a component of me where I feel like my faith drives a lot of my choices and, you know, we just pray for peace and protection, each day and, move forward in that way”. (FG 9: hesitant non-UTD)

Source notes: FG = focus group, NH = non-hesitant, UTD = up-to-date.

**Table 4:** Recommendations and strategies to improve trust, confidence, accessibility, and communication from parental focus groups 2022.

Advice to Share with Parents	
**Theme**	**Quotes**
Take ownership of decision making and carry out evidence-based research	“I would say, do your research on it and just, you know, if you get it for yourself, then why not get it for your child” (FG 9: hesitant non-UTD)
	“Make sure they’re listening to the right voices, like the voices of healthcare professionals and not just random people” (FG 9: hesitant non-UTD)
	“[I recommend to] continue to be a team player with their health care team, just so that you don’t run into any type of, you know, situations that you regret, whether it’s getting vaccinated or not getting back through” (FG 9: hesitant non-UTD)
Share personal experiences and listen with empathy	“What I generally say to parents is, listen, if you don’t want to get your baby vaccinated, and you want to take that risk it is your personal choice. But when, if you ever want to get some additional information, take a walk in the NICU, maybe your view will change. I’m not going to tell you what to do, but I can show you some things” (FG 7: hesitant non-UTD)
	“I like to ask them why, [and] what they believe to be the truth about the vaccine and why they’re hesitant, and seek to understand whether or not they trust their doctors, so I think that I try not to tell people anything and more focus on trying to elicit information from them” (FG 8: hesitant non-UTD).
Emphasize evidence that vaccination prevents serious harm	“As I’ve seen other people, you know, pass away from COVID, you know, I just want them, if you want your children to be safe, it is better to be safe and give them the vaccine than to be sorry and then have to let them go. No parent should bury their children” (FG 2: non-hesitant)
	“For people who are still as of yet, today, not vaccinated adults. I don’t go into these conversations about children. I’m not going to push my, what I think is right for my kid onto somebody else. I think it’s a very sensitive topic, but when it comes to like, just protecting my friends, you know, adults, I do try to emphasize that there is a tremendous amount of data available around the safety and effectiveness of the vaccines.” (FG 8: hesitant non-UTD)
Actions affect others	“I guess I would just tell them to keep your family safe and just don’t think about you. There’s others out here that we need to worry about as well. Like the elderly and children and such. So that’s what I would say to the parents: make a good wise decision. If you choose not to be vaccinated, just take precaution and that’s about it” (FG 8: hesitant non-UTD)
**Recommendations to Policy-Makers**	
**Theme**	**Quotes**
Make vaccination services and access to information convenient	“I think one of the ways that would probably be advantageous is to do like a mobile center where you take a van around and you go to areas that may be less vaccinated and just make it accessible” (FG 1: non-hesitant)
	“People generally want to call and get an answer right away…some way to get customer service so you have a question and a place to ask questions and get answers to your questions relatively quickly” (FG 7: hesitant non-UTD)
	“I think that there’s a lot cooking in the kitchen, but you can’t make people eat what you’re cooking or some people, but I can give you the tools to build a house [and] you [can] choose to build or to choose not to build, but I’m always going up there on the side of caution is for me and my family.” (FG 8: hesitant non-UTD)
	“I feel like all this, those things [convenient clinics open on nights and weekends] are done in different communities and different things is never going to be enough for some people.” (FG 8: hesitant non-UTD)
Leave politics out of policies	“When mask wearing became political, actually that to me was a little bit more frightening. I feel like with vaccines, that’s been an ongoing thing since vaccines have been around. And so, but mask wearing I’m like, that to me, I was just like, okay, we’re doing this for the community and that’s an easy thing, you know?” (FG 5: non-hesitant)
	“I would say stick to the facts and keep other politicians names and parties out of it so that our community health is not impacted because of, disagreements” (FG 9: hesitant non-UTD)
	“The transparency part and not being political, just with really being, humane, and just really caring about the people and understanding that [we] have lessons learned plan from this whole thing. So that if something new does come up again, politics are less out of it. And people’s wellbeing in is going to be the top priority” (FG 9: hesitant non-UTD)
Strive for transparency, honesty, and clarity	“I just want them to stop pushing this we’re going back to normal narrative because COVID changed the world so we’re not going back to normal, we’ve got to go forward to something new. We have to live in a new place” (FG 2: non-hesitant)
	“Listen, really listen to their [health professonal’s] recommendations and don’t make it a political issue. And I think with the messaging, make sure that the messaging reflects that this is not a cold or flu, but it’s an actual disease and it has long-term effects even for kids so that people understand the risks of choosing not to be vaccinated” (FG 9: hesitant non-UTD)
Accept that some will not choose vaccination	“See, so you fall on one side of the coin on the mandate that they did the right thing to mandate or you’re not sure. It’s difficult. It’s a hard question for me because it has pushed some of these people into a corner where they’re even more adamant about it” (FG 3: non-hesitant)
	“I feel like all this, those things [convenient clinics open on nights and weekends] are done in different communities and different things is never going to be enough for some people” (FG 8: hesitant non-UTD)
Consider carefully before removing incentives	“I feel like at this rate or what I’ve been seeing for other parents, or, I mean, my dad still has not gotten vaccinated, even though we’ve all told him, he can’t see his six grandchildren. So that always makes me hesitant where I’m like, I don’t know what more of an incentive there should be then seeing your grandchildren to get vaccinated…. I hope that like with parents and caregivers, especially of younger children, it comes down to access to things… Access to restaurants, concerts, traveling…. And I’m fearful that if those things like suddenly get lifted, which I think they’re starting to, it’s just going to backpedal, you know, I’m like, oh, dad let’s travel to Ireland, but you can’t until you’re vaccinated. I’m like, I hope that stays, you know, I don’t know if those types of policies shift” (FG 2: non-hesitant)
	“For policy makers, you know, personally, I also, I mean, I said, I feel safer in a place where vaccine mandates are required and I think that’s a better way to go about functioning in society” (FG 4: non-hesitant)
Leaders lead by example	“I felt some kind of comfort, even though it was a little cynical when you saw like, Fauci or other, you know, docs in the area, getting on TV, getting a shot. And even though you think, well, they could be getting anything in there, in that needle, but it did also make me feel more confident like these leaders, people telling us to get it” (FG 3: non-hesitant)
Listen to communities and recognize one size does not fit all	“I mean, for grown-ups there seems like there’s a lot of pluses to being vaccinated. Like for a long time, we were the only ones who could go to restaurants and things like that. But tell me how it, what’s in it for a seven-year-old, like how does his life get better? I don’t see there being any real policy implications for children…. So I would like there to be know that the Omnicom surge is over, throw us some kind of bone that will make our kids’ lives a little bit better” (FG 2: non-hesitant)
	“I think that what’s really important for policymakers to understand is that we’re all coming from different backgrounds. We’re coming from different communities and cultures where there might be misunderstandings. There might be a difference in the way that folks have been treated in the health system prior…. And that’s, you know, that helps me better understand how to be more respectful and more understanding of people who have different opinions about the vaccine than I do.” (FG 4 non-hesitant)
	“I think it would have been more effective to take a local approach. You know, have a national strategy, but implement it locally so that we’re not doing more harm than good in trying to get this, get the pandemic under control. [An example is] So like lockdowns and putting small business owners, you know, having people lose their lifelong work and say an area where COVID rates were very low, for example, and risk of transmission was very low. You know, also applying those [lockdown policies] fairly, you know, why is Target open, but the mom and pop hardware stores closed” (FG 8: hesitant non-UTD)
Learn from past lessons	“There is a certain amount that we do just have to mandate, right? We mandate seatbelts, we mandate, you know, not having, you know, pipes built with lead in them. You have to mandate things to keep people safe and so I think that our decisions about what we are doing should be really driven by the science and be mandated.” (FG 4: non-hesitant)

## Data Availability

Not applicable.

## Supplementary Materials

The following supporting information can be downloaded at: https://www.mdpi.com/article/10.3390/vaccines10081277/s1. Table S1: The Children’s Hospital of Philadelphia outpatient care network patient demographics, 2022. Figure S1: Sampling method for study recruitment for parental vaccine confidence survey and focus groups from the Children’s Hospital of Philadelphia (CHOP) outpatient primary care network, 2021–2022.
