# Supplementary material for: Factors Influencing Parental and Individual COVID-19 Vaccine Decision Making in a Pediatric Network
Source: Vaccines (Basel). 2022 Aug 8;10(8):1277. doi: 10.3390/vaccines10081277 (PMC9412825; doi:10.3390/vaccines10081277)
Supplement: Supplementary file 1 [file vaccines-10-01277-s001.zip › vaccines-1832819-supplementary.pdf]

vaccines MDPI

Table S1. Children's Hospital of Philadelphia Outpatient Care Network Patient Demographics, 2022

CHOP Site Type	Urban Academic N (%)	Urban Non-Academic N (%)	Suburban N (%)	Total N (%)
**Gender**				
Female	29,066 (49%)	15,234 (49%)	94,986 (49%)	139,286 (49%)
Male	29,790 (51%)	15,893 (51%)	99,002 (51%)	144,685 (51%)
Unknown	0 (0%)	0 (0%)	3 (0%)	3 (0%)
**Race**				
White or Caucasian	5462 (9%)	12,100 (39%)	125,915 (65%)	143,477 (51%)
Black or African American	42,416 (72%)	13,487 (43%)	22,350 (12%)	78,253 (28%)
Asian or Pacific Islander	2732 (5%)	1277 (4%)	9941 (5%)	13,950 (5%)
Multiple	1355 (2%)	1198 (4%)	5927 (3%)	8480 (3%)
Other or Unknown	6891 (12%)	3065 (10%)	29,858 (15%)	39,814 (14%)
**Ethnicity**				
Hispanic or Latino	5092 (9%)	2268 (7%)	16,273 (8%)	23,633 (8%)
Non-Hispanic or Latino	53,591 (91%)	28,516 (92%)	176,378 (91%)	258,485 (91%)
Unknown	173 (0%)	343 (1%)	1340 (1%)	1856 (1%)
**Patient Age**				
< 1 year	9979 (17%)	5663 (18%)	28,478 (15%)	44,120 (16%)
1-5 years	16,021 (27%)	8297 (27%)	49,147 (25%)	73,465 (26%)
6-8 years	9048 (15%)	4125 (13%)	29,053 (15%)	42,226 (15%)
9-12 years	11,560 (20%)	5538 (18%)	39,053 (20%)	56,151 (20%)
13-17 years	10,761 (18%)	6218 (20%)	42,219 (22%)	59,198 (21%)
18-19 years	1487 (3%)	1286 (4%)	6041 (3%)	8814 (3%)
**Payer**				
Medicaid	43,498 (74%)	8648 (28%)	42,268 (22%)	94,414 (33%)
Commercial or Other	15,296 (26%)	22,439 (72%)	151,430 (78%)	189,165 (67%)
Unknown	62 (0%)	40 (0%)	293 (0%)	395 (0%)

Source Notes: Academic sites have a higher proportion of uninsured and Medicaid patients, include physicians in training, and use different patient and provider quality metrics compared with the non-academic urban and suburban sites.

Figure S1. Sampling Method for Study Recruitment for Parental Vaccine Confidence Survey and Focus Groups from Children's Hospital of Philadelphia (CHOP) Outpatient Primary Care Network, 2021–2022.

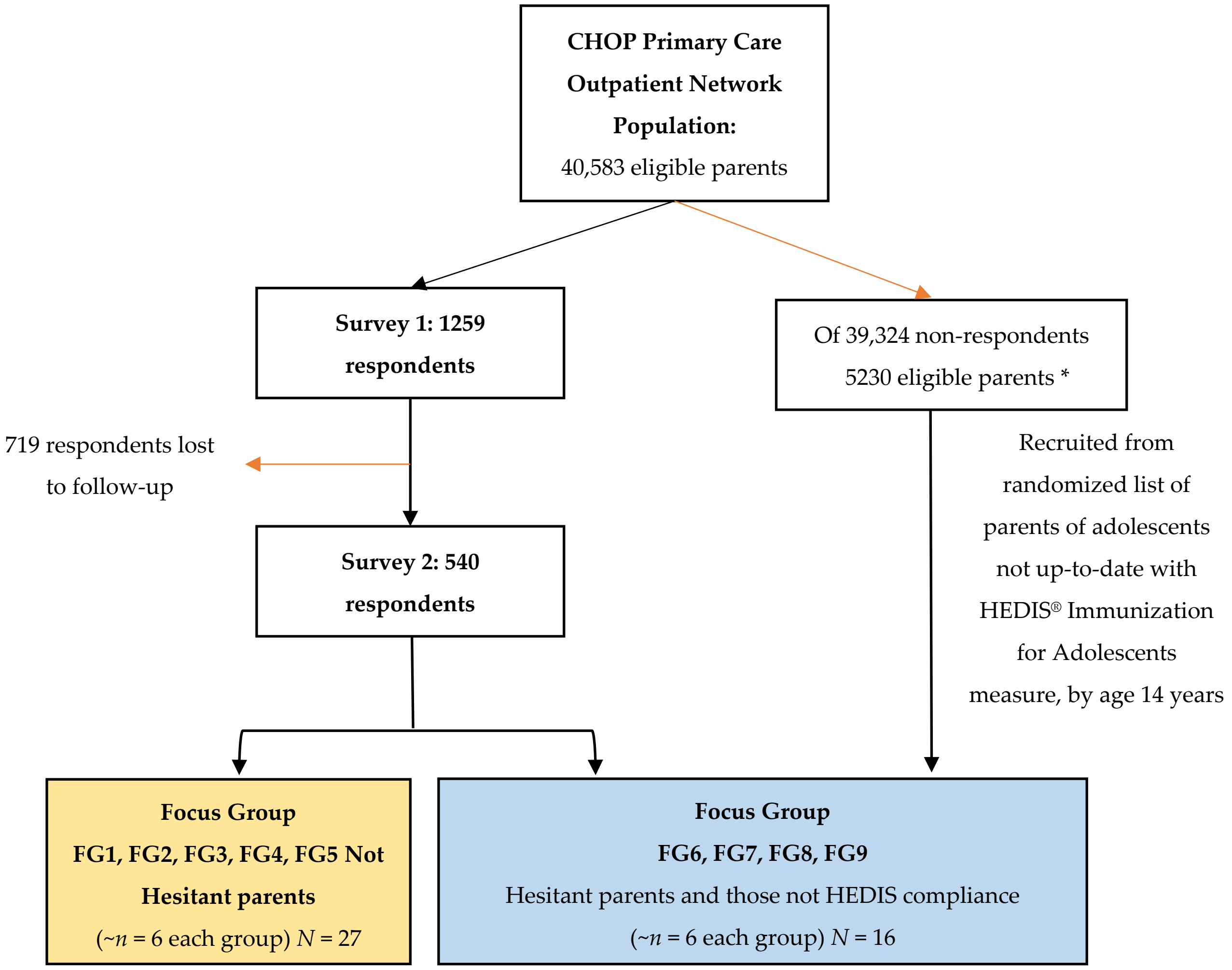

Note: Surveys one and two were conducted from October 12–January 21, 2022 and January 27–February 18, 2022, respectively. The unit of analysis is a parent. HEDIS® = Healthcare Effectiveness Data and Information Set. * Hesitancy is defined as per Helmkamp, et. al., as measured responses on a 4-point Likert scale (Strongly Disagree, Disagree, Agree, and Strongly Agree) resulting in the following numeric scores: 1, 2, 4, or 5. Some items were reverse-coded so a lower score indicated hesitancy. Hesitancy was defined as a mean score < 3 (the midpoint).[15] One dose of meningococcal vaccine, one tetanus, diphtheria, acellular pertussis (Tdap) or tetanus (TD) vaccine and the complete human papillomavirus vaccine (HPV) series by age 13 years. For the recruitment we extended the age of compliance to 14 years because of delays in care due to the COVID-19 pandemic.
